# When Distraction Holds Relevance: A Prospective Memory Benefit for Older Adults

**DOI:** 10.3390/ijerph120606523

**Published:** 2015-06-09

**Authors:** Joana S. Lourenço, Elizabeth A. Maylor

**Affiliations:** Department of Psychology, University of Warwick, Coventry CV4 7AL, UK; E-Mail: lourenco.joana@gmail.com

**Keywords:** prospective memory, aging, distraction, lures, inhibition

## Abstract

Evidence is accumulating to show that age-related increases in susceptibility to distracting information can benefit older more than young adults in several cognitive tasks. Here we focus on prospective memory (*i.e.*, remembering to carry out future intentions) and examine the effect of presenting distracting information that is intention-related as a function of age. Young and older adults performed an ongoing 1-back working memory task to a rapid stream of pictures superimposed with to-be-ignored letter strings. Participants were additionally instructed to respond to target pictures (namely, animals) and, for half of the participants, some strings prior to the targets were intention-related words (*i.e.*, animals). Results showed that presenting intention-related distracting information during the ongoing task was particularly advantageous for target detection in older compared to young adults. Moreover, a prospective memory benefit was observed even for older adults who showed no explicit memory for the target distracter words. We speculate that intention-related distracter information enhanced the accessibility of the prospective memory task and suggest that when distracting information holds relevance to intentions it can serve a compensatory role in prospective remembering in older adults.

## 1. Introduction

An essential aspect of cognitive functioning relates to the need to minimize or otherwise ignore environmental distraction that interferes with successful concentration on one’s current task. Distractions can come from various sources and be of different types, but it is generally assumed that distraction can disturb performance on the task at hand, such as when trying to read this paper in a noisy environment or during office hours with students coming in and out. Another indispensable aspect of a person’s everyday life is the capacity for remembering to remember, also termed prospective memory (PM). As remembering is frequently needed in contexts that can vary to a great degree, so can the challenges that each of these contexts poses to the individual’s capacity to interrupt the ongoing activity at the appropriate moment to fulfill the intended action. It is often the case that people are presented with multiple sources of information and must focus on the one that is relevant to their current goals.

In the present study, we focus on event-based PM, that is, tasks where a specific event occurring in the environment is the trigger to perform the prospective action [1]. The notion explored here is that some environmental distractions may hold more relevance than others, such that some of the ignored information might, at times, be of interest to the person’s future intentions and aid PM performance. For example, while you are engaged in browsing information on a topic that caught your attention at a seminar you attended early in the day, concurrent irrelevant information such as advertisements or email alerts might cause distraction. However, the sight of incoming messages might also remind you to send an email to a restaurant confirming table numbers for the evening meal, before you leave for a meeting that you have to attend shortly. In other words, the distracting email alerts were intention-related and therefore prompted you to subsequently perform your intended activity.

In laboratory-based PM studies, participants are typically asked to carry out an ongoing cognitive activity and also to perform an intended action when a particular event or PM target occurs (e.g., [1]). Research examining PM performance in the face of task-irrelevant information is scarce and, so far, has neglected the influence of aging. In the current study, we investigated whether presenting distracting information can benefit PM when this information is related to the intention. Furthermore, based on research showing older adults’ increased susceptibility to distracting information, we examined whether there are age-related differences in the potential benefit from distracting information on prospective remembering.

### 1.1. PM and Distraction

An important issue is how PM is affected by distraction. Research has typically focused on examining how PM performance is disrupted by distraction caused by demanding ongoing activities. A number of studies have shown that increasing the cognitive demands or processing requirements of the ongoing task can reduce PM performance (e.g., [2,3,4,5]). Although this line of research has resulted in important practical and theoretical contributions, these studies do not address effects of distraction caused by material that individuals are told to ignore and, particularly relevant here, what effects intention-related distracting information has on PM performance.

To our knowledge, the only study that has experimentally manipulated exposure to intention-related material in the to-be-ignored stream did so to examine if noticing of this material would occur and was limited to young adults. Specifically, Marsh *et al*. [6] (Experiment 1) instructed participants to perform a PM task of responding to visually presented animal words in the context of a pleasantness-rating task to visually presented words. In addition, participants were explicitly told to ignore words that would be concurrently presented in the auditory channel during the ongoing task. Critically, intention-related material (*i.e.*, animal words) was presented auditorially as to-be-ignored information and awareness for this material was examined in a subsequent recognition test for the words in the auditory stream. Results showed higher recognition memory for intention-related words compared to words from a control category. Marsh *et al.* [6] suggested that forming a categorical intention would heighten the category activation and likely bias attention towards to-be-ignored information that was related to the intention. Moreover, the authors showed that when the intention was not active during the pleasantness-rating task (because the intention was linked instead to a syllable-counting task to be performed later in the session), recognition memory for the intention words in the ignored channel no longer differed from memory for the control words.

Thus, results support the claim that having an intention can cause intention-related distracter material presented during the performance interval to be noticed and differentially processed. Furthermore, as pointed out by Marsh *et al.* [6], whether an attentional bias toward intention-related distracting information can result in a benefit for PM performance certainly merits clarification from future research. Importantly, previous research has shown that when participants are given a categorical PM task, presentation of words semantically related to the intention (*i.e.*, semantic lures) during the ongoing task can improve PM performance relative to a condition with no intention-related lures (e.g., [7]; see also [8]). Moreover, Kvavilashvili and Fisher [9] found evidence that, in naturalistic PM tasks, intentions are spontaneously retrieved by individuals from time to time as a result of encountering incidental external cues that are related to the intention. These continuous incidental triggers probably enhance the likelihood of fulfilling the intention by increasing the activation of the intention and further sensitizing the individual toward the occurrence of the target and/or relevant environmental events. These findings suggest that presentation of intention-related information as distracting material might benefit PM performance; however, to date this has not been examined. Moreover, previous research has not addressed the effect of aging on PM performance when distraction is related to the intention.

### 1.2. Distraction and Aging

Much prior work has examined how attention to information that is irrelevant to the task at hand can interfere with cognitive performance (e.g., [10,11]). Most relevant to the present investigation, several studies have focused on age-related differences in the susceptibility to distracting information. Results generally reveal that performance in a multitude of cognitive tasks is disproportionately affected by concurrent irrelevant information in older relative to young adults. For instance, older adults’ reduced ability to ignore irrelevant information results in performance deficits in processing speed tests [12], visual attention tasks (e.g., [13]), speech comprehension and reading (e.g., [14,15]), problem-solving [16], and episodic retrieval [17].

Hasher and colleagues’ inhibition theory assumes that older adults have reduced inhibitory control, or reduced ability to prevent irrelevant information from gaining access to attention/working memory [12,18,19,20]. The proposal is that a primary determinant of age-related differences in cognitive abilities is older adults’ impoverished capacity to efficiently regulate distraction. Moreover, recent evidence suggests that older adults’ increased distractibility may be due to reduced connectivity within the frontoparietal cognitive control network and consequent disruption of top-down attention [21,22].

However, whereas the focus of much research in aging and distraction has been on the negative effects of decreased attention regulation in older adults, more recently, interest in positive effects has arisen. In particular, several studies have shown that when distracting information becomes relevant in a subsequent implicit memory task, a benefit is often seen in older but not young adults (e.g., [11,23]; see also [24]). For example, Kim *et al.* [25] showed that reading stories that included distracter words that were solutions to a problem-solving task performed subsequently in the session increased the number of problems solved in older, but not young, adults. More recently, it was shown that implicit transfer of previously distractive information can also improve older adults’ free recall performance [17].

Of particular relevance here is a study on the positive effects of distraction in older adults [10] using a paradigm first developed by Rees *et al*. [26] to investigate inattentional blindness in young adults. In Rees *et al.*’s study, young participants were presented with a rapid stream of pictures appearing one at a time; their task was to monitor these pictures and to detect any immediate repetitions (*i.e.*, a 1-back working memory task). Superimposed on each picture was a to-be-ignored (distracter) letter string that was either a meaningful familiar word or a meaningless string of random letters. In a subsequent surprise recognition memory test for the meaningful words they had been shown, participants failed to differentiate between these distracter words and never-presented foil words. Moreover, fMRI data during the 1-back task showed that cortical activation for words was similar to that found when the superimposed strings were random letters. This was in clear contrast with the word- *vs.* non-word-related activation differences observed when participants in another condition were instructed to attend to the letter-strings stream instead of to the pictures stream. Thus, Rees *et al.*’s results are consistent with inattentional blindness for words presented in an attended location in young adults, such that there was no memory for the words nor was there any differential processing observed for words compared with random letters presented as to-be-ignored information.

Rowe *et al.* [10] adapted this paradigm to examine implicit memory for distracter words in young and older adults. Results showed increased completion of word fragments in older compared with young adults when the solutions matched previous distractive information in the 1-back task on pictures. Findings were therefore consistent with a performance advantage in older adults that follows from their poor attention regulation for irrelevant information.

### 1.3. PM and Aging

A number of laboratory-based studies have demonstrated robust age-related deficits in event-based PM tasks (e.g., [27,28,29,30]). In contrast, other studies have reported minimal or no age differences in prospective remembering (e.g., [31,32,33]). On the assumption that older adults have reduced attentional resources [34,35], it has been suggested that inconsistent findings might be the result of variations across studies in the level of strategic demands imposed by the tasks. In particular, more effortful tasks would have a deleterious effect on older adults’ performance (e.g., [36,37,38]). Consistent with this proposal, results from a meta-analysis [38] showed that age-related differences in PM are smaller for focal than for non-focal tasks (*i.e.*, where the target features associated with the intention are, or are not, part of ongoing-task processing, respectively) (but see also [39]).

Accordingly, in contrast to tasks high in strategic attentional demands, it is assumed that older adults are less penalized when successful PM can rely on spontaneous retrieval processes, which are hypothesized to be stimulus-triggered (e.g., [4,8,40]). Moreover, a recent experiment conducted by Altgassen *et al*. [41] showed worse PM performance for older than for young adults with neutral but not with emotionally valenced targets. The authors argued that emotional valence increased the salience of the targets. And, because salient targets facilitate involuntary capture of attention, and thus reduce the need for resource demanding processes, an increase in prospective remembering was observed.

### 1.4. The Present Study

Our main questions were whether presenting distracting information that is intention-related can lead to a PM improvement, and whether aging would influence the contribution of target distracting material to PM performance. We adapted Rees *et al.*’s [26] paradigm in which a 1-back task was performed on target pictures superimposed with to-be-ignored strings of letters. Specifically, a PM task was embedded in the 1-back task such that participants were also asked to give a PM response to pictures of animals. To examine whether intention-related distracting information would lead to a PM benefit, for half of the participants some of the distracter strings occurring before the PM targets were animal words. Our expectation was that presentation of target words as distracters might enhance the sense of familiarity for the targets and lead to a PM benefit due to an increase in the extent to which successful retrieval can rely on spontaneous processes. Furthermore, given age-related differences in susceptibility to distracting information, we hypothesized that presentation of intention-related distracter words should be advantageous to PM performance for older but not young adults. Thus, our specific predictions were that young adults would show no effect of intention-related distracting information on PM because of their ability to ignore the distracters, whereas older adults would show a benefit—hence our emphasis in the Results on the corresponding planned comparisons. Considering research suggesting that aging can pose additional challenges to the ability to successfully carry out PM tasks, and on the assumption that attentional resources decline with aging [34,35], we aimed to determine if older adults’ increased susceptibility to irrelevant information can serve a compensatory role when distracting information holds relevance to their future intentions.

## 2. Method

### 2.1. Design and Participants

The experiment was a 2 × 2 between-subjects design, with age (young, older) and lures (present, absent) as factors. Fifty-seven young adults (26 female) aged 18–28 years and 59 healthy older adults (34 female) aged 58–83 years took part in the experiment. Young participants were undergraduate students from Warwick University (UK) who volunteered in exchange for course credit or were paid £4 (approximately $6) for their participation. Older participants were members of a panel of volunteers recruited by local advertisements to join the Warwick Age Study and were paid £10 (approximately $15) as a contribution to their travel expenses. Within each age group, participants were randomly assigned to the lures present/absent conditions; however, data from one young and one older participant in the lures-present condition and two older participants in the lures-absent condition had to be discarded as described below, leaving 28 participants in each of the four cells of the design (see Table 1 for details).

**Table 1:** Means (and Standard Deviations) for Demographic Information and Tasks Performed During the Testing Session for Each Condition.

Measure	Young		Older	
	Lures	No Lures	Lures	No Lures
Age (years)	21.2 (2.4)	20.6 (3.1)	71.2 (5.8)	72.1 (5.9)
Mill Hill vocabulary score	20.6 (3.9)	19.3 (3.9)	25.4 (4.1)	24.4 (3.5)
Simon Task—Mean correct response time (ms)				
Congruent	420 (86)	407 (81)	529 (88)	516 (75)
Incongruent	457 (97)	449 (75)	604 (93)	590 (70)
Digit Span				
Forward	9.5 (2.1)	9.5 (1.7)	8.2 (2.0)	8.8 (2.1)
Backward	7.0 (1.2)	7.8 (2.0)	7.0 (1.8)	7.4 (2.0)
Pictures Task—Hit rate	0.89 (0.08)	0.94 (0.07)	0.83 (0.11)	0.84 (0.12)
Lexical Decision Task—Mean correct response time (ms)				
Animals	537 (117)	524 (81)	638 (100)	624 (91)
Controls	534 (83)	535 (99)	637 (98)	645 (113)
Non-lures	541 (68)	543 (76)	658 (86)	640 (90)
New words	542 (70)	548 (79)	656 (80)	644 (84)

Participants were tested individually in sessions lasting 40 to 50 min. The multiple choice part of the Mill Hill vocabulary test [42] was administered as a measure of crystallized intelligence. The results were consistent with the literature (e.g., [35,43]) with young participants scoring significantly lower than older participants, *F*(1, 108) = 46.35, *MSE* = 14.99, *p* < 0.001. There was neither a main effect of lures, *F*(1, 108) = 2.67, *MSE* = 14.99, *p* > 0.1, nor an interaction between age and lures, *F* < 1, suggesting that, within each age group, participants in the lures present/absent conditions were well matched in terms of vocabulary.

Two further cognitive tasks were administered to ensure that expected age differences were evident and that there were no differences in either age group between participants assigned to the lures and no lures conditions. The first was a Simon task, which measures the degree of interference from task-irrelevant spatial information on responses to task-relevant non-spatial information [44]. Speeded responses with the left/right hand were required on the basis of the direction of left-/right-pointing arrows that appeared on the left/right side of the screen. For accuracy, there was a significant Simon effect, with responses on congruent trials (e.g., left-pointing arrows on the left side of the screen) more accurate than on incongruent trials (e.g., left-pointing arrows on the right side of the screen) (97.0% *vs.* 92.4% correct, respectively), *F*(1, 108) = 40.15, *MSE* = 0.003, *p* < 0.001; there was no effect of age or of lures and no interactions. There was also a significant Simon effect for mean correct response times (RTs), with responses faster on congruent than on incongruent trials, *F*(1, 108) = 123.86, *MSE* = 1456.99, *p* < 0.001. Moreover, older adults showed a significantly larger Simon effect than did young adults, *F*(1, 108) = 11.64, *MSE* = 1456.99, *p* < 0.001, and this remained the case when general age-related slowing was taken into account by comparing young and older adults’ Simon effects as proportions of their overall RTs (10.1% *vs.* 15.2%, respectively), *F*(1, 108) = 5.46, *MSE* = 0.013, *p* < 0.05 (*cf.* [45,46]). However, there were no main effects or interactions involving lures, all *F*s < 1, indicating that within each age group, those randomly assigned to the lures present and absent conditions were equivalent on at least one indicator of inhibitory functioning.

The second cognitive measure was the digit span subtest from the Wechsler Adult Intelligence Scale [47]. This requires the immediate repetition of digit sequences of increasing length in the exact order presented (forward span) or in the reverse order (backward span). As expected, forward span was greater than backward span, *F*(1, 108) = 79.36, *MSE* = 2.16, *p* < 0.001, and young participants significantly outperformed older participants, *F*(1, 108) = 4.53, *MSE* = 4.83, *p* < 0.05, especially for forward span, *F*(1, 108) = 4.00, *MSE* = 2.16, *p* < 0.05. Again, there were no main effects or interactions involving lures (all *p*s > 0.1) indicating that within each age group those randomly assigned to the different conditions were equivalent in terms of short-term/working memory.

### 2.2. Materials and Procedure

**Figure 1:**
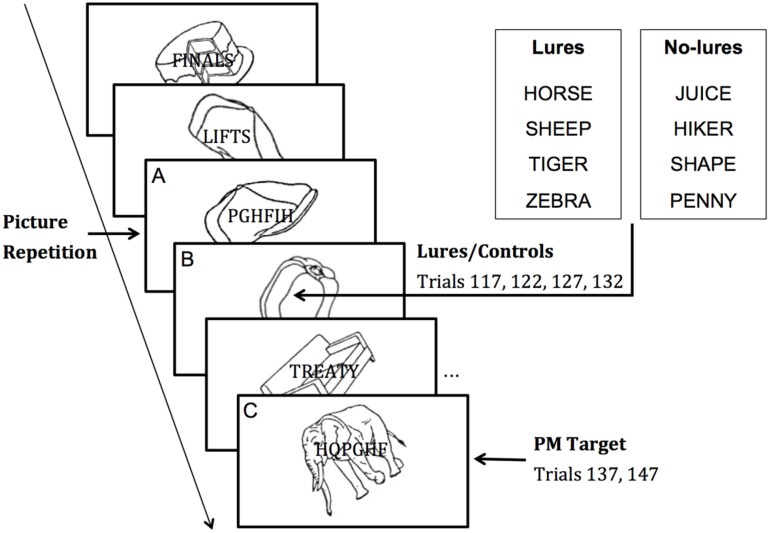
Schematic illustration of the experimental procedure for the 1-back pictures task. The ongoing task consisted of pressing the spacebar whenever two consecutive pictures were identical (**A**), while ignoring the strings of letters superimposed over each picture. The prospective memory (PM) task consisted of pressing the “B” key whenever a picture of an animal (**C**) was presented. Lure/control words were presented before Target 1 (**B**) according to the PM condition. Each picture-letters pair was presented for 1000 ms, followed by a 500-ms blank screen.

Participants were first given instructions about the 1-back visual working memory task (referred to as the “pictures” task; see Figure 1). They were presented with a rapid stream of individual pictures superimposed with either random letters or words. Participants were instructed to ignore the random letters/words and to press the spacebar whenever two consecutive pictures were identical. It was explained that the pictures could appear rotated, but that a correct response should be made even if the repeated picture was oriented differently. An example of a repeated picture presentation was then given and participants were informed that auditory feedback would be provided such that correct detection of picture repetitions would be followed by a bell sound and missed detections would be followed by a buzz sound (see [26]). Finally, participants were additionally given the PM instructions stating that if they ever saw a picture of an animal they should press the “B” key. Following encoding, participants were asked to explain the instructions to the experimenter and any omissions or mistakes were corrected. On each trial, the picture and letters pair was presented for 1000 ms, followed by a 500-ms blank screen. In addition, on consecutive picture trials, auditory feedback (*i.e.*, bell or buzz) was added during the blank screen. Before performing the pictures task, participants carried out the digit span task, which served as a delay between the PM task instructions and the beginning of the pictures task.

For the pictures task, 129 line drawings were selected from Snodgrass and Vanderwart [48] such that only two were pictures of animals (*i.e.*, the PM targets). Drawings were presented in the center of the screen and superimposed with either uppercase random letters or uppercase word strings. The strings had a length of five or six letters and were distinct for all trials (total of 90 random-letter strings and 60 word strings). Words were generated from the Balota *et al.* [49] lexicon database, were 1–2 syllables in length, and had a log-transformed Hyperspace Analogue to Language (HAL) frequency between 6 and 10. The strings were presented in a font size of 24 pt, subtending a visual angle of approximately 12° × 2° with a viewing distance of 50 cm. The pictures were presented with a maximum visual angle of approximately 12° × 12°.

The pictures task was composed of three blocks, with each block comprising 50 trials, seven of which were picture repetitions (150 trials with 21 repetitions in total). Before the start of each block, a screen with the block number was displayed for 2000 ms. The lag between repeated pictures ranged from two to seven intervenient pictures. In order to increase task demands, pictures were rotated 30° clockwise or counterclockwise from their natural axis and pictures had different orientations within any repeated pair (see [26]). Within each block of 50 trials, eight pictures with random letters superimposed were followed by 42 pictures with either random letters (22 in total) or words (20 in total) superimposed. PM targets and lures were only presented during Block III as described next. There were two PM target pictures presented on trials 137 and 147 (elephant and mouse, respectively). In addition, in the lures present condition, four of the superimposed words were animal words, presented on trials 117, 122, 126, and 132 (horse, sheep, tiger, and zebra, respectively). In the lures absent condition, the animal words were replaced by control words (juice, hiker, shape, and penny) that were matched with the lures on number of letters, syllables and mean HAL frequency. Of the seven picture repetitions in Block III, there were three picture repetitions before the first lure/control word, one after the first, third and fourth lure/control words, and finally one between the PM targets.

Following the pictures task, participants performed a lexical decision task (LDT) in which words from the pictures task were included. Participants were instructed to decide as quickly and accurately as possible whether a string of letters formed a word or not by pressing the right (“M”) key with their right index finger if the string was a word, or the left (“Z”) key with their left index finger if it was not a word. Next, participants were given 20 practice trials and the opportunity to ask questions before they commenced the task consisting of 10 buffer trials and 240 lexical decision trials (half words and half non-words). The word trials consisted of the four animal, four control and 56 non-lure words used in the pictures task and of 56 new words matched with the former on number of letters, syllables and mean HAL frequency according to Balota *et al.* [49]; non-words were selected from the same source and were 5–6 letters in length. Each trial consisted of a fixation cross presented for 500 ms, followed by the letter string presented at the center of the screen until the participant responded or 4000 ms had elapsed, and finally a 1000-ms blank screen.

At the end of the LDT, participants answered a post-experiment questionnaire to test their recall of the PM task as well as awareness of the animal lures presented as distracter words in the lures condition. Specifically, participants were asked to repeat the full instructions for the pictures task and memory for the PM task was checked. Then participants were asked if they noticed any animal words during the pictures task and, whenever they answered yes, participants were asked to list the animal words they remembered seeing. Next, they performed the Simon task and, finally, participants completed a demographic questionnaire and the Mill Hill vocabulary test before being debriefed.

## 3. Results

When queried about the PM task during the post-experiment questionnaire, one young and three older participants had no memory for the PM target/action and so their data were not included due to their failure in encoding and retaining the instructions. For all analyses, the alpha level was set at 0.05 for inferring statistical significance. Estimates of effect size (*η*_p_^2^) of significant effects are reported.

### 3.1. PM Performance

**Figure 2:**
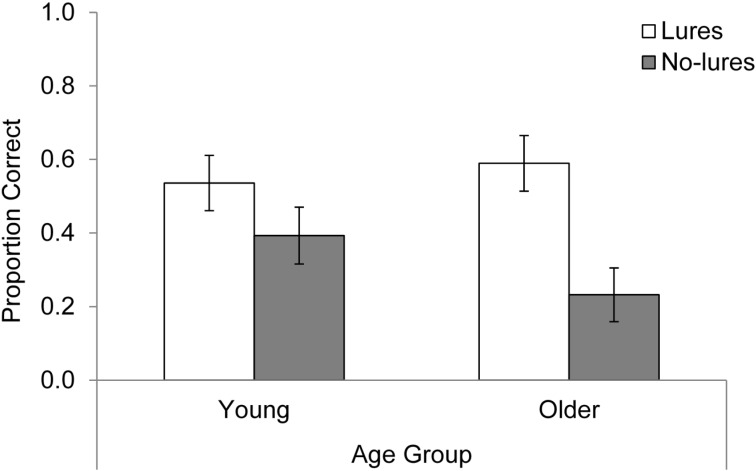
Mean proportion correct for the PM task across conditions. Error bars represent ± 1 standard error.

PM performance was scored as the proportion of target pictures for which the participant pressed the “B” key during the presentation of the target or within the next two trials. Ninety-six percent of the PM responses occurred during these periods. The overall means are shown in Figure 2. A 2 × 2 ANOVA with age (young, older) and lures (present, absent) as between-subjects factors revealed a significant main effect of lures, *F*(1, 108) = 11.88, *MSE* = 0.15, *p =* 0.001, *η*_p_^2^ = 0.10, such that target detection was better in the lures than in the no lures condition. Thus, although participants were told to ignore the letter strings superimposed on the drawings, there was a benefit in PM performance when some of these strings were intention-related (*i.e.*, animal words). The main effect of age was not significant, *F* < 1, and the interaction between age and lures failed to reached significance, *F*(1, 108) = 2.18, *p =* 0.143. Still, our planned comparisons revealed what is apparent in Figure 2, which is that the benefit of intention-related words was largely limited to older adults, *t*(54) = 3.52, *p* < 0.001. For young adults, there was little difference as a function of the presence or absence of intention-related material, *t*(54) = 1.38, *p* = 0.174.

Because multiple PM observations may not be independent as practice effects can occur across trials [27,50], the processes involved in trials other than the first PM target might obscure interesting effects. Therefore, and in line with the use of this approach in previous research (e.g., [4]), we examined if the effect of intention-related distracting material was more prominent on the first trial by conducting a binary logistic regression analysis of responses only to the first PM target (success *vs.* failure). Age (young, older) and lures (present, absent) were entered as categorical variables together with the interaction between them. The overall model was significant, 2Log-likelihood = 145.21, Nagelkerke R^2^ = 0.09, and there was a significant contribution to the prediction of PM success from the age by lures interaction, Wald’s χ^2^(1) = 5.26, *p* = 0.022, Exp(B) = 0.161 (95% confidence interval = 0.034–0.766). Again, intention-related distracter words benefited the PM performance of older adults (17/28 succeeded with lures; 7/28 succeeded with no lures; χ^2^(1) = 7.29, *p* < 0.01), but failed to benefit the PM performance of young adults (11/28 succeeded with lures; 13/28 succeeded with no lures; χ^2^(1) = 0.29). There were no other significant effects.

Finally, we examined mean PM success when the samples in the lure conditions were composed solely of participants with no explicit memory for the intention-related distracter words (*i.e.*, excluding participants who, during the post-experiment questionnaire for the PM task, could recall at least one animal word and had, therefore, noticed the presence of animal words in the distracter stream (ten young adults and seven older adults in the lures condition noticed the presence of distracter animal words and were therefore not included in the analysis)). A 2 × 2 ANOVA with age (young, older) and lures (present, absent) revealed a main effect of lures, *F*(1, 91) = 5.17, *MSE* = 0.14, *p* = 0.025, *η*_p_^2^ = 0.05, that was qualified by a significant age by lures interaction, *F*(1, 91) = 5.40, *MSE* = 0.14, *p* = 0.022, *η*_p_^2^ = 0.06. As before, the presentation of intention-related words improved target detection for older adults (*M* = 0.60, *SD* = 0.37, for lures, and *M* = 0.23, *SD* = 0.37, for no lures, *t*(54) = 2.84, *p* = 0.006), but not young adults (*M* = 0.39, *SD* = 0.37, for lures, and *M* = 0.39, *SD* = 0.39, for no lures, *t* < 1). Thus, for participants with no explicit memory for the target distracter words, a benefit was observed for older, but not young, adults. This replicates the pattern observed both for mean PM performance and PM performance for the first target.

### 3.2. Ongoing Task Performance

Proportion of picture repetitions correctly detected (hit rate) was computed (ongoing task false alarms, *i.e.*, spacebar key presses to pictures that had not been presented in the trial immediately preceding, were very rare and are thus not discussed further), and included in a 2 × 2 × 3 ANOVA with age (young, older) and lures (present, absent) as between-subjects factors, and block (first, second and third) as the within-subjects factor. There was a main effect of block, *F*(2, 216) = 3.50, *MSE* = 0.02, *p* = 0.032, *η*_p_^2^ = 0.03, such that hit rate was lower in the first (*M* = 0.85, *SD* = 0.17) than in the second (*M* = 0.89, *SD* = 0.15) and third (*M* = 0.89, *SD* = 0.12) blocks. The only other significant effect was a main effect of age, *F*(1, 108) = 17.30, *MSE* = 0.03, *p* < 0.001, *η*_p_^2^ = 0.14, such that hit rate was higher for young (*M* = 0.91, *SD* = 0.08) than for older (*M* = 0.83, *SD* = 0.12) participants. Thus, older adults were performing worse on the 1-back ongoing task than young adults.

### 3.3. Lexical Decision Performance

Immediately after the pictures task, participants performed a LDT involving words from the pictures task. Lexical decision accuracy was very high at 97% correct for both words and non-words. Based on previous research (e.g., [51]), RTs to non-lures and new words were trimmed to include only correct responses that were less than 2.5 standard deviations away from each participant’s mean. Trimming was done separately for the two types of trials and resulted in the elimination of 2.5% of RTs. Mean correct RTs (in ms) in the LDT were included in a 2 × 2 × 2 mixed ANOVA with age (young, older) and lures (present, absent) as the between-subjects factors, and word trial (non-lure, new) as the within-subjects factor. This revealed only a main effect of age, *F*(1, 108) = 50.69, *MSE* = 12,428.14, *p* < 0.001, *η*_p_^2^ = 0.32, such that as expected older adults produced longer RTs than young adults (see Table 1).

Next, we included RTs in a 2 × 2 × 2 mixed ANOVA with age (young, older) and lures (present, absent) as the between-subjects factors, and word trial (animal, control) as the within-subjects factor. There was only a main effect of age, *F*(1, 108) = 37.78, *MSE* = 15,892.67, *p* < 0.001, *η*_p_^2^ = 0.26, such that again older adults produced longer RTs than young adults. The absence of a main effect or any interactions involving word trial suggests that, similar to what has been shown for PM targets [52], there is no evidence of an intention interference effect (*i.e.*, slowing to intention-related material) in a LDT following completion of the PM task.

Finally, we examined RTs for non-lure words in more detail by conducting a 2 × 2 × 3 mixed ANOVA with age (young, older) and lures (present, absent) as the between-subjects factors, and non-lures location in the pictures task (Block I, II, or III) as the within-subjects factor. Again, the main effect of age was significant, *F*(1, 108) = 49.63, *MSE* = 19,370.72, *p* < 0.001, *η*_p_^2^ = 0.32. There was also a main effect of non-lures location, *F*(1, 216) = 11.24, *MSE* = 128,943.22, *p* < 0.001, *η*_p_^2^ = 0.09. *Post-hoc* analysis using the LSD test showed that RTs decreased from Block I (603 ms) to Block II (595 ms) to Block III (587 ms). Thus, there appears to be some evidence of repetition priming for the words previously superimposed in the pictures task, which was similar across age groups. However, the effect appears to be short-lived as facilitation in the processing of non-lure words appears to be dependent on recency of the initial presentation of this material.

## 4. Discussion and Conclusions

Our primary goal was to examine if the presentation of intention-related material as distracting information differentially impacts young and older adults’ PM performance. The findings provide the first evidence that intention-related distracting information presented during an ongoing task is particularly advantageous in enhancing target detection in older compared to young adults. Moreover, the PM benefit shown for older adults was not the result of noticing the target distracter words. In particular, an improvement was observed even for participants who showed no explicit memory for this material.

The present study establishes that a benefit of intention-related material on PM performance can be observed even though this material is presented as distracting information, that is, information that is irrelevant to the task currently being performed. Importantly, results further suggest that the benefit from intention-related distracting material is limited to older adults. Specifically, we found that PM performance was significantly higher when intention-related distracter words were presented, compared to control words, in older but not in young adults. The pattern of results was similar for mean PM performance and performance to the first target only (*i.e.*, performance that is independent of success to previous target occurrences). And although the lure by age interaction did not reach significance for mean PM performance, planned comparisons confirmed that a benefit was present for older but not young adults. Moreover, the benefit was found in the absence of differences for ongoing task performance between the lures and no lures conditions for each age group. Notably, findings from the present research showing that intention-related distracting information can facilitate older adults’ PM performance converge with results demonstrating an age-related benefit of poorer distraction control. In particular, previous research suggests that older adults’ reduced inhibitory control [12,18,19,20] can benefit older adults’ performance when distracting information becomes relevant in a subsequent implicit memory task (e.g., [10,11,25]). For example, Rowe *et al.* [10] showed that exposure to target pictures with to-be-ignored superimposed words increased the performance of older, but not young, adults in a subsequent word fragment completion task when the solutions had appeared as the distracter words in comparison to when the solutions had not appeared earlier. Thus, the findings from the present study align well with research on distraction control to suggest that older adults’ performance is more likely to be influenced by distracting information than that of young adults. Importantly, we extend those findings to the area of PM, which has been associated with age-related declines in performance.

When target rather than control distracter words were presented, older adults’ PM performance not only increased but also reached a similar level to that observed for young adults (see Figure 2). On the assumption that older adults have reduced attentional resources [34,35], it has been suggested that minimal age-related impairments in PM are expected when successful performance can rely on stimulus-triggered or spontaneous retrieval processes (e.g., [4,8,40]). In the present study, what processes might have been facilitated by the inclusion of intention-related distracter words to support PM performance? Prior findings of an age-related benefit from distracting information have been mostly linked with an effect of implicit knowledge on performance (e.g., [25]; see also [17]). For instance, Campbell *et al.* [53] showed that, after participants performed a 1-back task to target pictures with distracter words superimposed, using preserved picture-word pairs in a paired-associates memory task improved older but not young adults’ performance. Notably, the authors showed that this differential transfer of distraction in older compared to young adults was observed even though participants showed no explicit memory for the picture-word pairs. Accordingly, of further interest in the present study was the pattern of PM performance in the intention-related distraction group for those participants who reported no memory for the target distracter words. Importantly, we found higher target detection for these participants than for those presented with control words, and the benefit on PM performance was again limited to older adults.

Although reliant only on indirect evidence about the participants’ awareness of intention-related distracter words, our results suggest that for the majority of older adults the target distracter words were processed and led to a PM benefit, even though they failed to be consciously perceived. These findings are in line with the claim from the multiprocess view [4,40] that successful PM performance can rely on both resource demanding and spontaneous retrieval processes. According to this view, spontaneous retrieval can occur through either reflexive-associative or, more relevant here, discrepancy-plus-search processes. Within the discrepancy-plus-search view, it is assumed that when a PM target occurs, it may be processed more fluently or generate a sense of familiarity due to effects such as priming. This can stimulate search for the source of the discrepancy, which then results in recognition of the target as one that requires a PM response. Thus, recognition occurs in the absence of monitoring processes taking place prior to the occurrence of the target event [4,54].

Considering older adults’ reduced ability to inhibit irrelevant information, we suggest that the presentation of target words as distracters in the present study caused the representation of the intention to be strengthened in memory in older adults, and facilitated noticing of the PM targets presented a few trials later by boosting cue accessibility (*cf.* [22]). Noticing of target events as a result of cue-focused discrepancy attribution processes should be particularly advantageous for older adults given that the efficiency of the noticing component of prospective remembering is negatively influenced by age [55]. Moreover, the current results parallel those of a recent study showing that emotionally salient targets can eliminate age-related differences on PM performance. It was proposed that the effect was obtained because salience facilitates capture of attention and decreases the need for resource demanding processes [41,56]. Furthermore, previous research suggests that, provided that multiple intention-related events occur during the delay period and that these are fully processed, these items might trigger periodic thoughts about the PM task and increase the activation of the intention (e.g., [9,57]). This might be particularly crucial for older adults, given their increased susceptibility to momentary lapses of attention [58].

Importantly, Biss *et al.* [23] recently showed that exposure to distraction can also eliminate age-related differences in free recall. In three experiments, Biss *et al.* [23] had young and older adults study and recall a list of words on an immediate as well as on a surprise delayed recall test. Critically, in the delay participants completed a 1-back working memory task similar to the ongoing task in the present study (*i.e.*, a rapid stream of target pictures superimposed with to-be-ignored strings of letters), in which half of the studied words appeared as distracters. Biss *et al.* [23] found that older adults, but not young adults, showed reduced forgetting of the previously studied words that were presented as distracting information during the 1-back task. By contrast, the typical age-related difference was found in the recall of unrepeated words. Furthermore, Biss *et al.* [23] (Experiment 2) found that older adults responded more slowly on trials in which the distracters were the previously studied words relative to trials in which control words appeared. The authors suggested that exposure to distraction may serve as a rehearsal episode for older adults and improve memory by reactivating, or helping to keep accessible, that information. As noted by Biss *et al.* [23], boosted performance on the surprise delayed recall test due to older adults’ reduced ability to suppress distracting information occurs in the absence of intentions to rehearse, which is consistent with a benefit due to implicit rehearsal of distracting information [17,53].

It is interesting to note, however, that although our method greatly discouraged processing of the information presented in the distracter stream, at least some older and a few more young adults recalled seeing intention-related distracting information. This implies that at least on some proportion of the trials a few participants failed to ignore the target words. Although, at first, our results might appear contradictory with those of Rees *et al.* [26], it is important to note that we expanded their paradigm in several critical ways including embedding a PM task. In brief, Rees *et al.* [26] reported behavioral and fMRI data in young adults consistent with inattentional blindness for words presented in the attended location as to-be-ignored information. However, the authors presented each picture-letters pair in the pictures task for only 500 ms. With older adults in mind, and similar to Rowe *et al.*’s [10] study using the pictures task to examine distraction control in older adults, we used a duration of 1000 ms which probably caused changes in the demands posed by the 1-back task in comparison to Rees *et al.*’s [26]. As argued by the authors, incidental processing of lexical properties of the word stimuli may occur under task conditions that impose a lower load than the one created by the task parameters used in their study. Thus, as suggested both by memory for some of the target words in a few participants and the short-lived priming effect for distracter words evident in the LDT, it is possible that stimulus presentation time in the present study played a role in allowing occasional processing of letter strings.

Research has also shown that conscious attention can modulate the processing of task-irrelevant stimuli, such that a stimulus semantically congruent with an attended category is easier to detect than an incongruent stimulus. It is assumed that the effect is the result of a decrease in the detection threshold for category members due to attention directed to the attended category (semantic congruency effect; [59,60]). Importantly, in contrast with the present study and research on age-related distraction control, the previous findings were observed when presentation of the irrelevant stimulus was unexpected and limited to a single occurrence.

Two other points deserve mention. First, the present participants were asked to perform a non-focal PM task of responding to pictures of animals. Note that the task is considered non-focal (or “task-inappropriate”; [27]) because processing the pictures for the 1-back ongoing task does not require encoding of their category [61]. As mentioned in the Introduction, non-focal tasks generally yield age-related deficits in PM performance (e.g., [37]), but here there was no significant difference between young and older adults in mean target detection. Nevertheless, we found both that results were in the direction of a reduction, and that older adults performed worse than young adults in the ongoing task, with an accuracy of 83% (compared with 91%) for the detection of picture repetitions. Thus, it is possible that older adults maintained PM performance at the expense of greater costs to the ongoing task. In line with this notion, McDaniel *et al*. [62] showed that older adults can sometimes perform at similar levels to young adults in non-focal PM tasks by trading off performance on the ongoing task.

Second, the present study did not include a sensitive index of ongoing task costs, which would have required not only measuring response times to every ongoing trial but also a baseline condition without a PM task. We were therefore unable to determine the extent to which effortful monitoring, rather than cue-driven, processes occurred, and whether monitoring levels differed between age groups. Conceivably, the occurrence of related lures could have triggered periodic thoughts about the PM task and stimulated monitoring for the target events during critical points of the PM task (*i.e.*, in close proximity to the target events [8]). Nonetheless, although we used a non-focal target event, which would tend to encourage monitoring, we delayed the first PM target until near the end of the last of the three blocks of ongoing task trials, which would tend to discourage monitoring [63]. Moreover, a PM benefit was observed for older adults with no explicit memory for the target distracter words.

Research addressing alternative explanations for the present findings in terms of monitoring would therefore be an interesting avenue to pursue in the future. For example, one intriguing possibility is that monitoring may be disengaged until participants spontaneously retrieve the PM task in response to the occurrence of related lures, which could re-engage monitoring processes (*cf.* the dynamic multiprocess framework [64], which proposes an interplay between cue-driven spontaneous retrieval and effortful strategic monitoring processes that can be utilized dynamically to support PM performance). However, this would only occur in those who are inefficient in ignoring the unattended stream (e.g., older adults). Regardless, the present findings represent an important first step on the topic of (relevant) distraction in PM and its age-related benefit.

In summary, our experiment provided an opportunity to examine how age-related reduction in distraction control can affect PM when material related to the intention occurs in the ongoing task as information that participants are told to ignore. We have demonstrated for the first time a greater PM benefit from intention-related distracting information in older compared to young adults. However, the data obtained in the current study cannot precisely inform the nature of the processes that resulted in an age-related benefit in PM. Based on age-related differences in distraction control and attentional processes, we speculate that intention-related distracting information enhanced the accessibility of the PM task in older adults, leading to an increase in performance through spontaneous retrieval processes. Nonetheless, future studies are needed to determine the specific mechanisms that support PM improvement from distraction in older adults. In addition, evidence from a study by Gopie *et al.* [24] suggests that when the ability to engage in controlled processes is reduced, for instance by dividing attention with a secondary task, young adults show the same benefit from implicit memory for irrelevant information as do older adults. Thus another interesting direction for future work would be to investigate if these participants can similarly benefit from target distracting information to improve PM performance. Finally, the present findings have important real-world relevance as they suggest that, when distracting information holds relevance to the person’s intentions, older adults’ increased susceptibility to irrelevant information can serve a compensatory role and aid prospective remembering.
